# Selective Recovery of Flavanone-Enriched Fractions from Glycyrrhiza Glabra Leaves by Supercritical CO_2_ Extraction with Neuroprotective Potential

**DOI:** 10.3390/antiox15070874

**Published:** 2026-07-14

**Authors:** Simona Serio, Alessia Lambiase, Valentina Santoro, Anna Lisa Piccinelli, Rita Celano, Giorgia Spandri, Farida Tripodi, Luca Campone, Stefania Pagliari, Paola Coccetti, Cristina Solana-Manrique, Nuria Paricio, Mariateresa Russo, Massimo Labra, Luca Rastrelli

**Affiliations:** 1 Department of Pharmacy, University of Salerno, 84084 Fisciano, Italy; sserio@unisa.it (S.S.); vsantoro@unisa.it (V.S.); rcelano@unisa.it (R.C.); rastrelli@unisa.it (L.R.); 2 PhD Program in Drug Discovery and Development, University of Salerno, 84084 Fisciano, Italy; 3 National Biodiversity Future Center (NBFC), 90133 Palermo, Italy; a.lambiase1@campus.unimib.it (A.L.); farida.tripodi1@unimib.it (F.T.); luca.campone@unimib.it (L.C.); stefania.pagliari@unimib.it (S.P.); paola.coccetti@unimib.it (P.C.); massimo.labra@unimib.it (M.L.); 4 Department of Biotechnology and Biosciences, University of Milano-Bicocca, 20126 Milano, Italy; g.spandri@campus.unimib.it; 5 Department of Genetics and University Institute of Biotechnology and Biomedicine, University of Valencia, 46100 Valencia, Spain; cristina.solana@uv.es (C.S.-M.); nuria.paricio@uv.es (N.P.); 6 Department of Physiotherapy, European University of Valencia, 46100 Valencia, Spain; 7 Food Chemistry, Safety and Sensoromic Laboratory (FoCuSS Lab), Department of Agriculture Science, University of Reggio Calabria, 89124 Reggio Calabria, Italy; mariateresa.russo@unirc.it

**Keywords:** agricultural by-products, flavanone, responsible consumption and production—SDG12, good health and well-being—SDG3, lifespan, aging, protein aggregation, *Saccharomyces cerevisiae*

## Abstract

*Glycyrrhiza glabra* leaves (GGL) are an underutilized by-product of the licorice supply chain and a valuable source of bioactive flavanones. In this study, supercritical CO_2_ fluid extraction (SFE-CO_2_) was optimized for the selective recovery of leaf-exudate flavanones (pinocembrin, licoflavanone, and glabranin). Response surface methodology combined with UHPLC–UV profiling enabled the identification of optimal conditions, yielding high-purity (31% flavanones), solvent-free, and ready-to-use enriched extracts. SFE-CO_2_ outperformed solvent-based liquid extraction in terms of selectivity and enrichment capacity. Path2Green assessment indicated an environmentally sustainable and scalable profile. The extract exhibited antioxidant and anti-aging effects in a yeast model of Parkinson’s disease (PD), extending lifespan and reducing oxidative stress. Pinocembrin, the most abundant flavanone, inhibited α-synuclein aggregation in vitro and ameliorated PD-related phenotypes in a *Drosophila melanogaster* model, improving locomotor performance and increasing cellular energy metabolism. These results support the sustainable valorization of GGL into flavanone-enriched extracts with potential nutraceutical applications for aging-related disorders.

## 1. Introduction

*Glycyrrhiza glabra* L. (Fabaceae family) is a perennial species native to the Mediterranean areas [1]. Scientific and commercial interest has traditionally focused on roots and rhizomes (licorice), widely used as natural sweeteners and flavoring agents in the food, tobacco, and pharmaceutical industries, as well as herbal remedies for their expectorant, gastroprotective, and anti-inflammatory properties [1,2]. Among the different varieties, Calabrian licorice (*G. glabra* var. *typica*) is particularly valued for the quality and balanced sweet–bitter profile of its extracts [3], leading to the recognition of Protected Designation of Origin (PDO) “Calabrian Licorice” by the European Commission.

Besides roots, *G. glabra* develops a vigorous aerial biomass, which is emerging as a source of bioactive compounds with health-promoting and plant-protective properties. *G. glabra* leaves (GGL) and their constituents exhibit relevant antimicrobial, anti-inflammatory, antioxidant, α-glucosidase-inhibiting and anti-aging properties [4,5,6,7,8,9,10,11] as well as biocontrol activity against plant diseases [12,13,14]. Consistent with these biological properties, GGL displays a distinct profile of specialized metabolites compared to roots, including flavanones, dihydrostilbenes, and flavonol-O-glycosides [3,15,16]. Notably, GGL are particularly enriched in pinocembrin and prenylated flavanones (up to 16 g/100 g of dry leaf), and prenylated dihydrostilbenes (up to 7.5 g/100 g) [4,9], mainly accumulated in leaf-exudates [3]. These exudates are involved in ecological processes, contributing to plant defense against biotic and abiotic stresses [3,17,18]. Interestingly, we reported that GGL extracts collected during the blossom period are rich in exudate compounds like pinocembrin, licoflavanone, and glabranin and act as potent antioxidants, significantly reducing intracellular ROS levels, α-synuclein toxicity, and cellular senescence in the yeast *Saccharomyces cerevisiae*. These findings suggest GGL extracts are a promising natural agent for mitigating aging and age-related disorders [4]. Despite this evidence, GGL are still regarded as agricultural by-products, and are generally discarded during root harvesting, resulting in loss of valuable biomass [4]. The valorization of this untapped biomass could therefore improve sustainability of the licorice production chain, increase the value of PDO Calabrian licorice, and support sustainable development goals (SDGs) related to waste reduction and sustainable agri-food processes.

The effective exploitation of GGL requires safe and environmentally friendly extraction strategies able to reduce solvent use, processing time, waste generation, and environmental impact, while ensuring extract quality [19,20]. Among the most promising green extraction techniques, supercritical CO_2_ fluid extraction (SFE-CO_2_) is particularly suitable for recovering hydrophobic bioactive compounds from leaf surface exudates. CO_2_ is inexpensive, inert, non-toxic, and an easily recyclable solvent. SFE-CO_2_ enables procurement of solvent-free extracts of high-quality suitable for food applications, compared with conventional techniques. Moreover, SFE-CO_2_ represents a scalable green technology that can be readily integrated into a biorefinery approach [21,22].

In this context, this study aimed to develop an SFE-CO_2_ procedure for the selective recovery of exudate flavanones from GGL with zero solvent consumption and minimal co-extraction of matrix-components. SFE-CO_2_ optimization was performed through response surface methodology (RSM), monitoring extract composition by UHPLC–UV analysis, and the optimized process was compared with conventional solid–liquid extraction using organic solvents. Finally, the biological activity of the optimized extract and its major component, pinocembrin, was evaluated in experimental Parkinson’s disease (PD) models, focusing on protein aggregation, oxidative stress, and cellular energy homeostasis.

## 2. Materials and Methods

### 2.1. Chemicals and Standard

MS-grade acetonitrile (MeCN) and water were supplied by Romil Ltd. (Cambridge, UK) through Deltek S.r.l. (Deltek, Naples, Italy). Analytical-grade chloroform, ethanol, and hexane, MS-grade formic acid, 6-hydroxy-2,5,7,8-tetramethyl-chromane-2-carboxylic acid (Trolox), 2,2’-azinobis (3-ethylbenzothiazoline-6-sulphonic acid) diammonium salt (ABTS), 2,2′-azobis(2-methylpropionamidine) dihydrochloride (AAPH), fluorescein sodium salt, potassium persulfate (K2S2O8) and quercetin were obtained from Merck Chemicals (Milan, Italy). Pinocembrin (Pin), licoflavanone (Lic), and glabranin (Gla) were previously purified by preparative RP-HPLC from *G. glabra* exudate [3].

### 2.2. Samples

Leaves of *Glycyrrhiza glabra* var. typica (GGL) were supplied by Nature Med S.r.l. (Castrovillari, Italy) of the Consortium for Protected Designation of Origin “Liquirizia di Calabria PDO’’. Leaves were from a typical PDO production area in Castrolibero (Cosenza; coordinates (lat, long): 39.322460, 16.192634, 290 m a.s.l.) during the flowering stage in 2023 from 3- or 4-year-old plants. Leaves were oven-dried at 50 °C to constant dry weight to ensure stable moisture content (6–9%), determined by gravimetric analysis. The dried leaves were then ground using a Grindomix GM 200 knife mill (Retsch, Haan, Germany) and sieved to standardize particle size distribution between 300 and 600 µm. The resulting GGL material was stored under vacuum until use.

### 2.3. UHPLC–UV Quantitative Analysis

Quantitative analysis of GGL extracts was performed using an Ultimate 3000 UHPLC system coupled to an RS variable wavelength detector (Thermo Fisher Scientific, Germering, Germany). Chromatographic separation was performed using a Kinetex C18 column (2.1 × 100 mm, 2.6 μm; Phenomenex, Torrance, CA, USA) protected by a C18 Guard Cartridge (Phenomenex) and thermostated at 25 °C. A binary gradient of H_2_O and MeCN, both containing 0.1% formic acid, was used at 500 µL min^−1^ (5 μL injection volume) using the following elution program: 0–3 min, 2% B; 3–5 min, 2–13% B; 5–9 min, 13% B; 9–12 min, 13–18% B; 12–13 min, 18% B;13–17 min,18–30% B; 17–20 min, 30% B; 20–30 min, 30–40% B; 30–38 min, 40–60%B; 38–39 min, 60–98% B; 39–44 min, 98% B. UV spectra were acquired at 290, 220, and 350 nm, and flavanones were quantified at 290 nm. Flavanone levels in GGL extracts were determined by the external standard method. A mixture of reference standards (Pin, Lic, and Gla, 1 mg mL^−1^) was diluted with MeOH/H_2_O 1:1, *v*/*v* to prepare calibration, analyzed in triplicate. Calibration curves were linear over 1–200 µg mL^−1^ (R^2^ values > 0.998, ANOVA-tested). Intraday repeatability showed coefficient of variation (CV) values < 4%, indicating good method precision. GGL extracts were analyzed at 500 µg mL^−1^ and the amount of target compounds was expressed as g 100 g^−1^ extract (mean ± standard deviation, SD).

### 2.4. Supercritical Carbon Dioxide Fluid Extraction (SFE-CO_2_)

SFE-CO_2_ was performed using a Spe-ed Helix supercritical fluid extractor (Applied Separations, Allentown, PA, USA), operating in static and dynamic modes and equipped with 24 mL stainless steel cells and 60 mL collection bottles. The extraction cells were fitted with cellulose filters at both the top and bottom and loaded with the sample mixed with glass wool and 4 mm glass beads in a 1:2:2 (*w*/*w*/*w*) ratio. CO_2_ was used as the extraction solvent with a flow rate of 2 L min^−1^, and the CO_2_ needle heater was set at 80 °C. After each run, residual CO_2_ was vented by closing the inlet valve and opening the outlet valve; complete depressurization required approximately 10 min.

For the preliminary experiments involving variations in pressure and temperature, a single extraction cycle was applied, consisting of a 20 min static phase followed by a 90 min dynamic phase. Extracts were collected at 30 min intervals to monitor the extraction kinetics. Two sample loadings (1.2 and 2.4 g) were evaluated, and the lower one was selected to minimize raw material consumption. Under optimal conditions, SFE-CO_2_ was performed at 40 °C, 370 bar, and 30 min dynamic time. After extraction, the extraction yield (EY) was determined gravimetrically, and an aliquot of each extract was dissolved in ethanol and diluted with MeOH/H_2_O 1:1, *v*/*v*, to a final concentration of 500 µg mL^−1^ for UHPLC analyses.

### 2.5. Experimental Design and SFE-CO_2_ Optimization

SFE-CO_2_ conditions were optimized by Response Surface Design using a Box–Behnken design quadratic model (6 degrees of freedom), consisting of one block replicates of 16 randomized experimental runs and four center points. The experimental design and optimization were performed using Statgraphics Centurion 18 software (Statgraphics Technologies, Inc., The Plains, VA, USA). The independent factors (A, temperature; B, pressure; C, dynamic time) and their levels (−1, 0, and +1) are reported in Table S1. Extraction efficiency (EE, g 100 g^−1^ leaf) and extract content (P, g 100 g^−1^ extract) of Pin, Lic, and Gla were considered as response variables to be maximized. Furthermore, extraction yield (EY, g extract 100 g^−1^ leaf), total flavanone (Fs) contents in leaf and extract, and CO_2_ consumption were included as response variables. The experimental design conditions and the corresponding response variable values are listed in Table S1.

A second-order polynomial equation was used to fit each dependent variable (Y) as a function of the independent factors. Mathematical models of the estimated response surfaces were defined after removing the non-significant factors and their interactions identified by ANOVA (*p* > 0.05). Model adequacy was evaluated using the lack-of-fit test, while model quality and predictability were assessed through the regression coefficients (R^2^ and adjusted R^2^). Subsequently, a multi-response optimization of factors A–C was performed using the desirability function, aiming to maximize both EEs and Ps of target compounds. To prioritize Fs enrichment, a greater weight was assigned to the extract Fs content. The predicted optimal conditions were experimentally validated (*n* = 4) by comparing the predicted response values with the experimental results (95% confidence interval).

### 2.6. Exhaustive Extraction

Exhaustive extracts of GGL were achieved by ultrasound-assisted extraction using a Labsonic LBS2 ultrasonic thermostatic bath (FALC Instruments, Treviglio, Italy) [3]. Extractions were conducted in triplicate at 30 °C for 30 min using 70% (*v*/*v*) aqueous ethanol and solid–liquid ratio of 50 g L^−1^. Filtered supernatants (Whatman No. 1 filter paper) from three extraction cycles were pooled, and ethanol was removed under vacuum at 40 °C using a rotary evaporator (Rotavapor R-200, Buchi Italia S.r.l, Cornaredo, Italy). The remaining aqueous residues were then freeze-dried (Alpha 1–2 LD freeze dryer, Martin Christ Gefriertrocknungsanlagen GmbH, Osterode am Harz, Germany). The extraction yield was 38.9 ± 1.5 g extract per 100 g of dry matrix. The content of Pin, Lic, and Gla in the exhaustive extract were 6.0 ± 0.6, 4.6 ± 0.5, and 1.3 ± 0.1 g 100 g^−1^ extract, respectively.

### 2.7. Conventional Solvent Extraction

Maceration was employed as conventional solvent-based solid–liquid extraction (SLE) technique using chloroform and hexane as extraction solvents. An amount of 5 g of GGL in 100 mL of solvent was subjected to maceration at room temperature for 24 h. Extraction was conducted in triplicate. After extraction and filtration (Whatman No. 1 filter paper), organic solvents were removed under vacuum at 30 °C in a rotary evaporator. Dried extracts were treated as reported in Section 2.4.

### 2.8. Antioxidant Capacity (AOC)

The antioxidant capacities (AOCs) of GGL extracts were determined using the ABTS radical scavenging and oxygen radical absorbance capacity (ORAC) assays, as previously described [23]. All measurements were performed using a Varioskan LUX multimode microplate reader (Thermo Fisher Scientific, Vantaa, Finland). Quercetin was used as positive control, and Trolox calibration curves (2.5–25 μM and 0.5–7.5 μM, well concentrations, for ABTS and ORAC assays, respectively) were used to calculate AOCs. Results were expressed as Trolox equivalent antioxidant capacity (TEAC) per g of extract (mmol TE g^−1^, mean ± SD). In the ABTS assay, the tested concentration ranges from 40 to 650 μg mL^−1^ for exhaustive and SFE-CO_2_ extracts, and from 100 to 1000 μg mL^−1^ for chloroform-SLE extracts. For the ORAC assay, exhaustive and SFE-CO_2_ extracts were tested in the range of 0.2–1 mg mL^−1^, and chloroform-SLE extracts in the range of 0.25–1.25 mg mL^−1^.

### 2.9. Yeast Strains and Culture Conditions

The yeast strain BY4742 [pYX242-SNCA], overexpressing human α-synuclein, was used to evaluate the biological effects of SFE-CO_2_ extract and pinocembrin (Pin). Cells were cultured as reported in Lambiase et al., 2026 [24]. SFE-CO_2_ extract was suspended in 75 µL of ethanol, vortexed, sonicated for 20 min at 28 kHz, and added to 5 mL of culture medium at a final concentration of 0.2% *w*/*v*. Pin was dissolved in ethanol, vortexed, sonicated for 20 min, and added to 5 mL of culture medium at a final concentration of 150 µM. Control cultures contained 1.5% ethanol.

### 2.10. Chronological Lifespan (CLS) Assay and Intracellular Reactive Oxygen Species (ROS)

Yeast cells were grown in liquid medium to the mid-to-late exponential growth phase and then inoculated at 0.150 OD mL^−1^ into fresh medium containing 1.5% ethanol (control) or 0.2% SFE-CO_2_ extract or 150 µM Pin. Cell survival was assessed at selected time points by propidium iodide (PI) staining and flow cytometry using a CytoFLEX cytofluorimeter (Beckman Coulter, Brea, CA, USA). Data were analyzed with CytExpert software (version 2.6.0.105), as previously described [24]. Intracellular ROS levels were measured after 24 h using dihydroethidium (DHE) staining following an established protocol [24]. Fluorescence was measured by flow cytometry using the CytoFLEX cytofluorimeter.

### 2.11. Thioflavin T (ThT) Aggregation Kinetic Assay

For fibril elongation kinetic experiments, 20 µM α-syn (100 µL) was incubated at 37 °C, with 1 µM preformed seed fibrils and 20 µM ThT in PBS, up to 72 h under constant shaking (425 cpm) using a BioTek Synergy H1 plate reader (BioTek Instruments, Winooski, VT, USA) in the absence (control) or presence of Pin (10 and 100 µM). Pin was dissolved in EtOH, which was also used as solvent control.

Seed fibrils were prepared by incubating 250 µL of 70 µM α-syn in PBS at 37 °C for 72 h under constant shaking (900 rpm) in a thermo-mixer. Fibril formation was verified through the ThT assay by mixing 10 µL of fibril samples with 20 µM ThT in a final volume of 100 µL. Fluorescence emission at 485 nm was recorded using a BIoTek Synergy H1 plate reader.

### 2.12. Drosophila Culture and Drug Administration

*DJ-1β*^ex54 mutant flies (*DJ-1β*), used as a *Drosophila* model of PD [25], were maintained on standard fly food at 25 °C. Second instar (L2) *DJ-1β* larvae were reared on standard food containing 0.1% DMSO (control) or supplemented with Pin at concentrations of 100 or 200 µM. After eclosion, adult female flies were transferred to fresh vials containing the same treatments and maintained for 5, 10, and 15 days. Flies were then subjected to climbing assays or frozen in liquid nitrogen and stored at −80 °C for biochemical analyses.

### 2.13. Climbing Assays and Lifespan Analyses in Flies

Motor performance was evaluated using a negative geotaxis climbing assay [26]. Female flies numbering 80–120 *DJ-1β*, cultured either in control or Pin-supplemented food, were tested under two treatment regimens: during development (from larval stages to five days after eclosion) or only in adulthood. In both cases, groups of 10–20 *DJ-1β* female flies were placed in graduated tubes (1.4 cm diameter), acclimated for 1 min, gently tapped to the bottom, and given 10 s to climb. Climbing ability was expressed as the mean height reached per fly.

For lifespan experiments, 75 one-day-old *DJ-1β* female flies per condition were divided into groups of 25 and housed in vials containing either control food (0.1% DMSO) or Pin-supplemented food (200 µM). Mortality was recorded every 2–3 days, and flies were transferred to fresh food weekly until death. Survival curves were generated and analyzed using Kaplan–Meier plots and log-rank tests.

### 2.14. Protein Carbonylation Assay, ATP Quantification, and Glycogen Determination

Protein carbonyl content was quantified in extracts from groups of 10 5-day-old *DJ-1β* female flies using 2,4-dinitrophenylhydrazine (DNPH) derivatization in 96-well plates, as previously described [27].

ATP levels were measured using an ATP determination kit (Invitrogen, Waltham, MA, USA). Briefly, groups of five 5-day-old *DJ-1β* female flies, cultured either in control or Pin-supplemented food, were homogenized in 200 µL reaction buffer, boiled for 4 min, and centrifuged at 18,500× *g* for 10 min at 4 °C to remove debris. Subsequently, 5 µL of each surnatant was mixed with 100 µL of reaction solution in a white 96-well plate, and luminescence was recorded using an Infinite 200 PRO plate reader (Tecan, Männedorf, Switzerland). Results were expressed as relative luminescence units per mg of proteins and normalized to control samples.

Glycogen content was measured following an adapted protocol [28]. Groups of five 5-day-old *DJ-1β* female flies, cultured either in control or Pin-supplemented food, were homogenized in 200 µL PBS using a TissueLyser LT (Qiagen, Hilden, Germany) with a steel bead (2 min, 50 Hz) and centrifuged at 180× *g* for 10 min at 4 °C. Then, 90 µL of supernatant was mixed with 10 µL of 20% (*w*/*v*) sodium sulfate and 750 µL ethanol. After vortexing, samples were centrifuged (180× *g*, 15 min, 4 °C) to precipitate glycogen. Pellets were washed twice with 80% methanol, resuspended in 1 mL anthrone reagent (1.42 mg mL^−1^ in 70% sulfuric acid), and incubated at 90 °C for 15 min. After cooling and centrifugation, 200 µL of each sample was transferred to a 96-well plate, and absorbance was measured at 625 nm using an Infinite 200 PRO reader.

### 2.15. Statistical Analysis

The quantitative data and TEAC values of GGL extracts were analyzed by one-way ANOVA (*p* < 0.05), followed by Tukey’s HSD test for multiple comparisons.

All biological experiments were performed at least in triplicate. Data are presented as mean ± SD. Statistical significance was evaluated using two-tailed Student’s t-tests (*p* < 0.05). For *Drosophila* experiments, comparisons among groups were evaluated using one-way ANOVA followed by Tukey’s post hoc test (*p* < 0.05).

## 3. Results

### 3.1. Selective Recovery of GGL Flavanones by SFE-CO_2_

CO_2_ is widely employed in SFE due to its readily accessible supercritical conditions (31.1 °C and 74 bar) and green chemistry compliance, as it is non-toxic, non-flammable, and classified as generally recognized as safe (GRAS) for food applications [20,29,30]. Under supercritical conditions, CO_2_ exhibits high diffusivity, while its solvent power and density can be finely tuned by adjusting the temperature and pressure. Extraction performance strongly depends on these parameters [20,29,30]. The effectiveness and the quality of the process therefore rely on optimizing these parameters, along with other factors such as extraction time, sample loading, matrix moisture content, and the use of modifier, to maximize target compound recovery while preserving extract integrity [20,29,30]. In the present study, the residual moisture content of the dried GGL was below 10%, a level generally considered suitable for SFE-CO_2_, as excessive moisture may hinder CO_2_ diffusion and mass transfer through the plant matrix and can lead to co-extraction of water, thereby affecting extraction efficiency and extract handling [22,30].

#### 3.1.1. Preliminary SFE-CO_2_ Experiments

**Figure 1:**
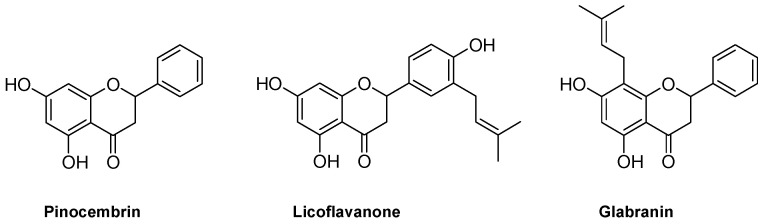
Main flavanones of *Glycyrrhiza glabra* leaves.

Preliminary extraction trials were conducted to define key parameters, narrow the experimental domain, and identify the most relevant factors for chemometric optimization. Experiments were conducted in duplicate under fixed conditions (20 min static phase, 90 min dynamic phase), with aliquots collected every 30 min to monitor extraction kinetics. Results were expressed as amount of Pin, Lic, Gla (Figure 1), and total flavanones (Fs) determined by UHPLC–UV analysis. These trials established the operating limits for temperature, pressure, and sample loading.

**Figure 2:**
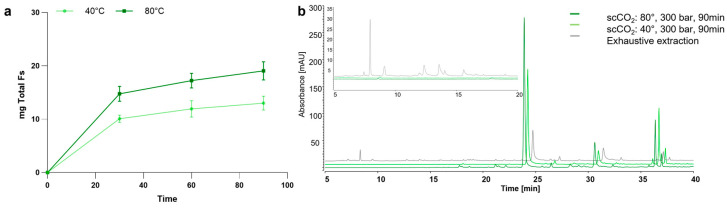
UHPLC–UV chromatograms (290 nm) of SFE-CO_2_ extracts at 300 bar and 80 °C (dark green) and 40 °C (light green), compared with exhaustive extract (grey) (**a**). Amount (mg) of total flavanones (Fs) at different temperatures (300 bar) as a function of extraction time (90 min) (**b**). Values are expressed as mean ± SD (*n* = 2).

Temperature between 40 and 80 °C have been used for extracting lipophilic bioactive compounds such as parthenolide from feverfew leaves [31], lycopene from tomato skin [32], and xanthohumol from hops [33], and cannabinoids [34]. In our study, increasing the temperature from 40 to 80 °C, at 300 bar, improved Fs recovery by 47% (Figure 2a), likely by enhancing CO_2_ diffusivity and mass transfer, while preserving the naturalness of the extract (Figure 2b). Accordingly, 80 °C was selected as the upper temperature limit.

**Figure 3:**
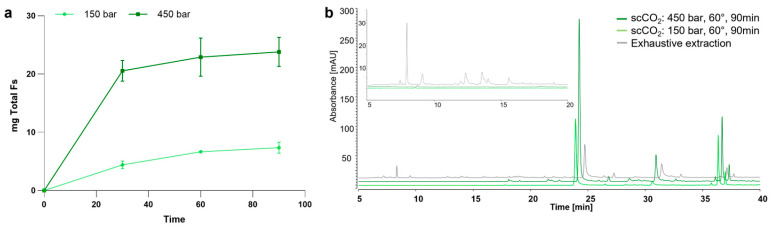
UHPLC–UV chromatograms (290 nm) of SFE-CO_2_ extracts at 60 °C and 450 bar (dark green) and 150 bar (light green), compared with exhaustive extract (**a**). Amount (mg) of total flavanones (Fs) at different pressures (60 °C) as a function of extraction time (90 min) (**b**). Values are expressed as mean ± SD (*n* = 2).

The pressure range (150–450 bar) was selected based on literature reports spanning from 100 bar for eugenol [35] to 450 bar for lycopene extraction [32], with intermediate values for xanthohumol (350 bar) [33], parthenolide (220 bar) [31], and cannabinoids (200–400 bar) [34]. At 60 °C, the Fs amount at 150 bar was 68% lower than that achieved at 450 bar (Figure 3a), confirming that higher pressure increases CO_2_ extraction efficiency. However, excessive pressure may reduce SFE-CO_2_ selectivity and affect pump performance reproducibility [36]. Thus, the maximum pressure was set at 400 bar to ensure process stability and efficient, selective recovery.

Regarding extraction time, preliminary experiments showed a typical SFE kinetic profile (Figure 2a and Figure 3a), with a constant extraction rate during the first 30 min, corresponding to rapid recovery of accessible metabolites, followed by falling-rate and diffusion-controlled stages (30–90 min), where mass transfer limitations increased [37].

Finally, sample loading was assessed using two matrix amounts (1.2–2.4 g, 24 mL extraction cell), giving comparable recoveries. A CO_2_ flow rate of 2 L min^−1^ was selected to ensure efficient extraction without needle freezing.

#### 3.1.2. Optimization of SFE-CO_2_ by RSM

Preliminary experiments confirmed the effectiveness of SFE-CO_2_ in selectively recovering the target metabolites (Pin, Lic, and Gla) from GGL. As shown in Figure 2b and Figure 3b, no glycosylated flavonoids from inner leaf tissue were co-extracted, confirming the intrinsic selectivity of SFE-CO_2_ toward hydrophobic flavanones.

Based on these findings, a response surface methodology (RSM) based on Box–Behnken design (BBD) was applied to evaluate the effects of temperature (A), pressure (B), and dynamic time (C) on SFE-CO_2_ performance and to identify optimal operating conditions. The selected response variables were extraction efficiency (EE, g 100 g^−1^ leaf), extract content of target flavanones (P, g 100 g^−1^ extract), and extraction yield (EY). The experimental domain (A: 40–80 °C; B: 150–400 bar; C: 30–90 min) and design matrix are reported in Table S1. Results (Table S1) showed that SFE-CO_2_ consistently produced extracts highly enriched in target Fs, with enrichment levels up to three-fold higher than exhaustive extraction. Extraction yields were comparable to those reported for SFE-CO_2_ without modifier [38,39].

**Figure 4:**
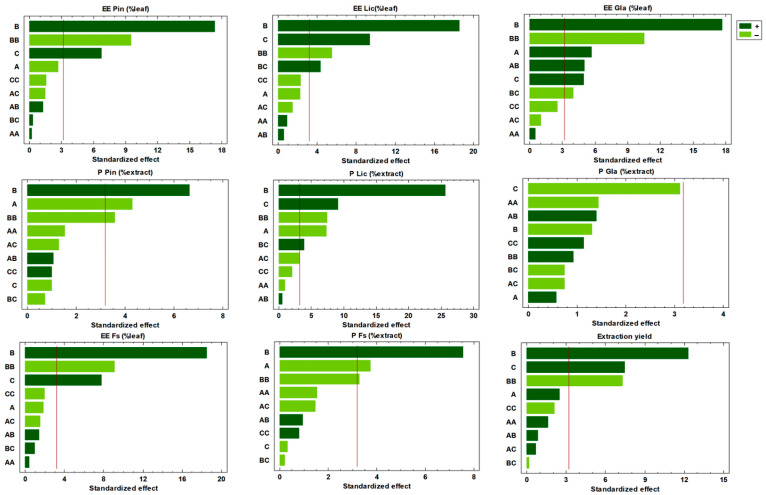
Pareto charts of standardized effects of SFE-CO_2_ factors on response variables. The vertical line marks significance at the 95% confidence level.

The statistical significance of factors A–C and their interactions was assessed by ANOVA (Table S2), and the standardized effects on response variables are shown in the Pareto charts (Figure 4). No statistically significant effects of factors A–C were observed for Gla content; therefore, this response was excluded from further chemometric analysis. Pressure (B) and its quadratic term (BB) emerged as the most influential factor for all remaining responses, whereas temperature (A) and dynamic time (C) showed selective effects. Temperature significantly affected only the extract content of Pin, Lic, and Fs, while dynamic time primarily impacted EEs and EY.

**Figure 5:**
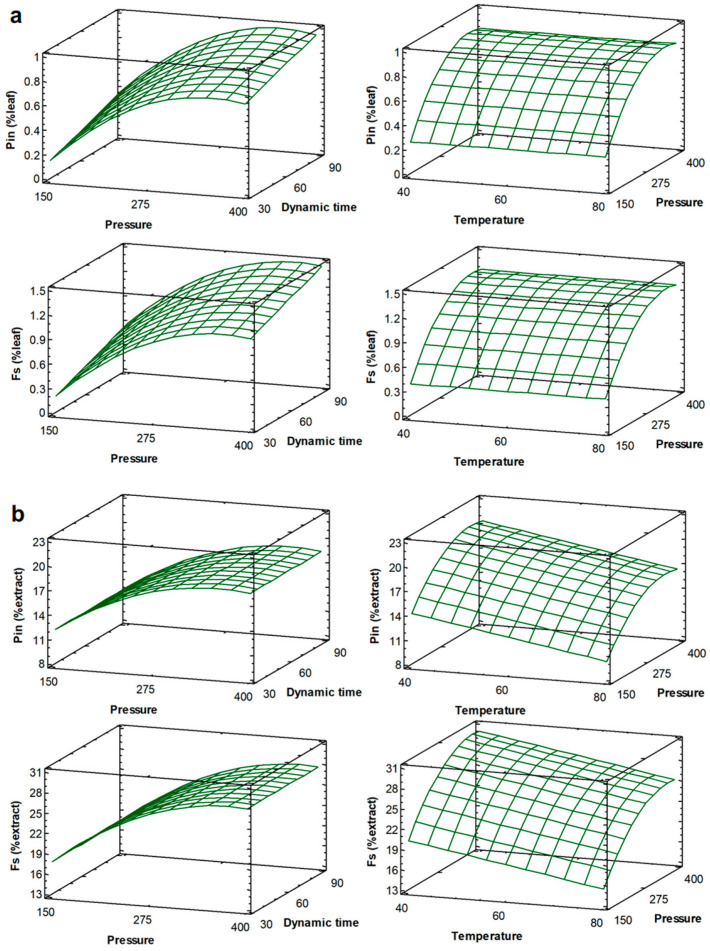
Response surfaces of (**a**) extraction efficiency and (**b**) extract content of Pin and Fs as a function of pressure and dynamic time (on the left, temperature fixed at 60 °C) and of temperature and pressure (on the right, dynamic time fixed at 60 min). Only statistically significant effects are shown.

The combined effects of factors A–C are displayed in the response surface plots (Figure 5 and Figure S1), generated from model equations resulting from the removal of non-significant terms (*p* > 0.05) (Table S3). The regression models were highly significant (*p* < 0.0001) and accurate (lack-of-fit, *p* > 0.05) for all response variables (Table S2), indicating good model adequacy. Moreover, adjusted R^2^ values (82–97%) indicated robust model predictability (Table S2).

As highlighted by the response surfaces (Figure 5 and Figure S1), pressure exerted the most pronounced influence on all responses. However, the negative contribution of its quadratic term (Figure 4) led to a plateau above 350 bar, indicating that further pressure increases do not enhance extraction performance. This is attributed to increased CO_2_ viscosity, reduced diffusivity, and enhanced co-extraction of nonpolar matrix components. Temperature showed a mainly negative linear effect on Pin, Lic, and Fs content (Figure 5 and Figure S1), with 40 °C as the optimal level for purity-related variables (Table S3). This trend is likely associated with enhanced co-extraction of matrix components at higher temperatures. In contrast, extraction efficiency was not significantly affected by temperature, except for Gla (Figure S1), the most non-polar metabolite. Dynamic time positively influenced EY and flavanone EEs, reaching maximum values at 90 min (Table S3), but did not significantly affect extract Fs content (Figure 5 and Figure S1). This is consistent with the surface localization of target metabolites, which are mainly recovered during the first 30 min (Figure 2b and Figure 3b). Longer extraction times increased the overall recovery but also promoted fluid saturation and co-extraction of lipophilic matrix components, reducing process selectivity.

Finally, a multiple response optimization was performed to simultaneously maximize both metabolite recovery and flavanone enrichment. Using the desirability function, the optimal conditions were identified as 40 °C, 370 bar, in 90 min (desirability index = 0.92). These conditions were experimentally verified, collecting extracts at 30 min intervals. The results revealed that by extending the dynamic time to 90 min the flavanone purity decreased by approximately 25% compared to the 30 min extract (Figure S2), while providing only a modest (~10%) increase in recovery.

As the aim of this study was to obtain enriched extracts of leaf-surface metabolites within a sustainable framework, a second optimization was conducted by including CO_2_ consumption as an additional response variable. By integrating this parameter, the optimal compromise between purity, recovery, and sustainability was achieved at 40 °C, 364 bar, and 30 min (desirability = 0.94) (Figure S3). Experimental validation produced results fully consistent with the 95% confidence interval of the predicted values (Table S1). Notably, this optimized protocol provided a highly enriched ready-to-use extract while reducing CO_2_ consumption to one third of the initial optimal settings.

#### 3.1.3. Sustainability and Performance of SFE-CO_2_ Compared with Conventional Methods

After optimization, SFE-CO_2_ performance was compared with solvent-based extraction (SLE). Ethanol and aqueous ethanol mixtures were excluded because of their low selectivity toward exudate flavanones (Figure 3a,b). Therefore, to ensure a meaningful comparison focused on selective recovery of target flavanones, solvents were selected based on their Hildebrand solubility parameters (δH), a suitable criterion for comparing SFE-CO_2_ with organic solvents [39,40]. Under optimized conditions (40 °C, 400 bar), SFE-CO_2_ exhibits a δH value of 16.6 MPa1/2, intermediate between hexane (14.8 MPa1/2) and chloroform (18.8 MPa1/2). These solvents were thus selected as benchmarks for extraction efficiency, enrichment capability, and sustainability.

As reported in Table 1, SFE-CO_2_ yielded extracts with the highest Fs content, approximately 3-fold and 16-fold more enriched in Fs than chloroform- and hexane-SLE extracts, respectively. Although chloroform-SLE achieved higher extraction efficiency (80% Fs recovery) and extract yield (35%), it produced extracts with substantially lower purity. This outcome reflects the limited selectivity of chloroform, due to the co-extraction of highly non-polar components such as waxes and lipids. Hexane-SLE showed the lowest performance in terms of yield, recovery, and extract purity (Table 1). The selective enrichment achieved by SFE-CO_2_ was also reflected in the antioxidant capacity of the extract evaluated using ORAC and ABTS assays. SFE-CO_2_ extract exhibited significantly higher AOC than CHCl_3_-SLE extract (Table 1) and GGL exhaustive extracts (ABTS, 3.4 ± 0.2 mmol TE g^−1^; ORAC, 4.7 ± 0.4 mmol TE g^−1^), with increases ranging from 27 to 42%. Notably, the higher antioxidant capacity was consistently observed in two assays based on complementary antioxidant mechanisms, supporting the effective enrichment of antioxidant constituents by SFE-CO_2_. This enhancement is likely associated with the three-fold enrichment in Fs and is consistent with previous evidence identifying exudate flavanones as the main contributors to GGL antioxidant activity [4]. Thus, SFE-CO_2_ not only selectively concentrates leaf-surface flavanones but also produces an extract with enhanced antioxidant capacity compared with both exhaustive and conventional solvent-based extraction.

From a sustainability perspective, SFE-CO_2_ showed clear advantages over solvent-based SLE according to the six principles of green extraction [19], particularly regarding solvent use, waste management, and processing time. Solvent selection should prioritize options that are biodegradable, non-persistent, and safe to handle in terms of flammability, volatility, and operator exposure [19,41]. Chloroform is classified as hazardous by the CHEM21 guide [42] because of toxicity and handling concerns. In contrast, CO_2_ represents a safer and sustainable alternative being non-toxic, non-flammable, and fully recyclable. Waste generation is also minimized: SFE-CO_2_ generates no liquid waste, and the residual biomass remains dry and solvent-free, allowing its direct reuse for further extraction or fractionation steps within a biorefinery-oriented strategy. Furthermore, under optimized conditions, SFE-CO_2_ produced high-purity extracts in approximately 30 min, compared with the 24 h required for conventional SLE.

**Figure 6:**
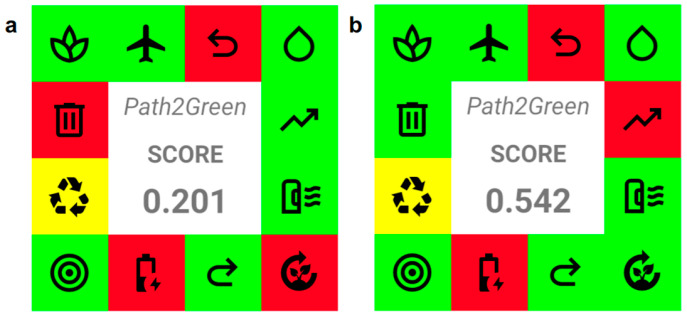
Path2Green pictograms of optimized SFE-CO_2_ at lab-scale (**a**) and large-scale (**b**). The surrounding tiles, proceeding clockwise from the top, correspond to the 12 principles of green extraction: (1) biomass, (2) transport, (3) pre-treatment, (4) solvents, (5) scaling, (6) purification, (7) yield, (8) post-treatment, (9) energy, (10) application, (11) repurposing, and (12) waste management. The color-coding indicates the degree of compliance: green (strong), yellow (moderate), and red (requiring improvement).

The SFE-CO_2_ process was specifically designed as a sustainable strategy to valorize underutilized GGL through the selective recovery of flavanones. Accordingly, an integrated sustainability evaluation was performed using the Path2Green metric, which evaluates environmental, economic, and social aspects across the entire extraction workflow, from biomass sourcing to process completion [41]. Based on the 12 Path2Green principles, the optimized SFE-CO_2_ achieved a final score of +0.201 (on a scale from −1 to +1) (Figure 6a), indicating a moderately sustainable profile. The main strengths included the use of waste from renewable local biomass (+1), a safe and recyclable solvent (+1), low impact of physical pre-treatment (−0.2), production of ready-to-use extracts (+1) without purification and post-treatment steps (+1), potential applications (+0.83), and good process reproducibility toward continuous extraction flow (+0.5) (Table S4). The limitations mainly concerned transport (+0.05), yield (−0.5), energy demand (−0.5), and waste generation (−0.9) (Table S4). Despite the high energy demand of SFE-CO_2_, the short extraction time and potential use of renewable energy can mitigate its environmental impact.

Although a detailed techno-economic assessment was beyond the scope of this study, the short extraction time, reduced CO_2_ consumption, and direct production of a solvent-free enriched fraction suggest that the higher capital cost of SFE-CO_2_ equipment could be partly balanced by lower solvent-related, waste-management, and downstream processing costs [19,22]. Importantly, the economic and environmental impact of the most critical parameters, namely transport, yield, and waste management, could be further mitigated through on-site valorization strategies, including installation of SFE-CO_2_ units within agricultural facilities (“hull technology”) combined with the reuse of residual biomass. As illustrated in Figure 6b, the large-scale plant would improve the Path2Green profile by addressing key limitations identified at laboratory scale, particularly those related to transport, repurposing, and waste management (Table S4) [41]. Indeed, the unexhausted matrix could be further exploited for the recovery of additional metabolite classes, improving the overall sustainability of the process within a biorefinery-oriented approach [22,41].

### 3.2. Neuroprotective Properties of SFE-CO_2_ Extract and Pinocembrin

#### 3.2.1. SFE-CO_2_ Extract and Pinocembrin Extend Longevity in a Yeast PD Model

**Figure 7:**
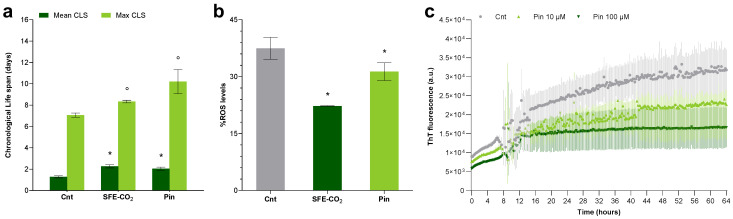
Effect of SFE-CO_2_ extract and pinocembrin (Pin) on *Saccharomyces cerevisiae* cells overexpressing human α-syn: (**a**) Chronological lifespan (CLS) assay of yeast cells in presence of 0.2% extract or of 150 μM Pin. Mean and maximum lifespan are shown as mean ± SD, *,° *p* < 0.05 relative to control cells. (**b**) ROS content of cells treated for 24 h as in (**a**), * *p* < 0.05 relative to control cells. (**c**) Kinetic of second nucleation of α-syn aggregation process, detected by ThT fluorescence, in the absence (cnt) or presence of Pin (10 or 100 μM).

We previously reported that GGL exhaustive extracts strongly attenuate α-syn toxicity, showing great potential to prevent aging and age-related disorders [4]. Remarkably, SFE-CO_2_ treatment increased both the mean and maximal lifespan and reduced the intracellular ROS levels compared to untreated cells, thereby maintaining the protecting properties against α-synuclein-induced aging and toxicity (Figure 7a,b). This is consistent with the high antioxidant capacity of the SFE-CO_2_ extract observed using ABTS and ORAC assays (Table 1) and, since oxidative stress is strongly associated with toxic protein aggregation induced by α-syn [43], it further supports the neuroprotective potential of this extract.

Since Pin is the most abundant compound of the SFE extract, its impact on the yeast PD model was investigated. When administered to α-syn-expressing yeast cells, Pin (150 µM) significantly extended the longevity of the yeast cells (Figure 7a), increasing both the mean and maximum lifespan by approximately 1.5- and 1.6-fold, respectively, compared with the control (Figure 7a). Moreover, a 24 h exposure to Pin reduced intracellular ROS levels by approximately 16% (Figure 7b).

To assess the direct effect of Pin on the protein aggregation, which represents a typical hallmark of PD, the Thioflavin T (ThT) assay was used. As protein aggregation involves three different steps, lag, exponential, and equilibrium phases, we analyzed whether Pin could influence fibril elongation by studying the aggregation of monomeric α-syn in the presence of preformed fibrils (PFF) that act as seeds for secondary nucleation. Pin inhibited fibril elongation in a dose-dependent manner, reducing fibril elongation compared to the control (Figure 7c). Indeed, incubation of monomeric α-syn and 1 µM PFF with 10 µM Pin resulted in a reduction of about 30% of fibril elongation, while Pin at 100 µM resulted in a stronger one, inhibiting fibril elongation of approximately 50%. These data clearly indicate that Pin has antiaggregant properties, influencing fibril elongation.

#### 3.2.2. Pinocembrin Suppresses PD-Related Phenotypes in a Drosophila Melanogaster Model

Given the well-established antioxidant and neuroprotective effects of Pin in various biological models [5,44] and its antioxidant effect on the yeast PD model, we assessed its impact in a *Drosophila* model of PD based on *DJ-1β* deficiency. DJ-1 is a key protein involved in cellular defense against oxidative stress [45], and *DJ-1β* mutant flies exhibit several PD-related phenotypes, including impaired locomotion, shortened lifespan, increased ROS levels, and susceptibility to oxidative stress [46]. This model is highly responsive to pharmacological treatments and has been extensively used to evaluate the efficacy of compounds with different mechanisms of action [47,48]. Notably, mutations in *DJ-1* cause autosomal recessive forms of PD, and oxidatively modified *DJ-1* protein accumulates in the brains of patients with sporadic PD [45].

**Figure 8:**
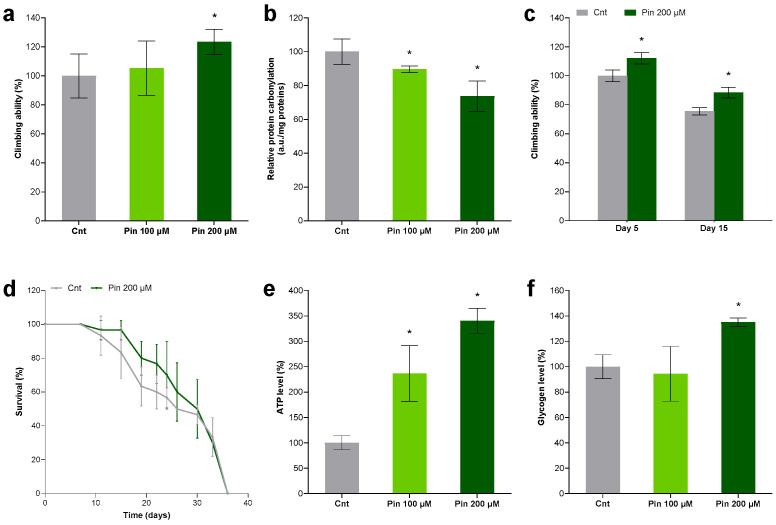
Effect of pinocembrin (Pin) on *Drosophila melanogaster* model of PD: (**a**) Motor performance of 5-day-old *DJ-1β* mutant flies treated with 100 or 200 μM Pin or vehicle (0.1% DMSO, Cnt) from larval stages was evaluated by a climbing assay. (**b**) Protein carbonylation levels in 5-day-old *DJ-1β* mutant flies treated with 100 or 200 μM Pin from larval stages. (**c**) Motor performance of *DJ-1β* mutant flies treated with 200 µM Pin only in adulthood was evaluated by performing a climbing assay at 5 and 15 days after eclosion (*n* = 80–120 female flies per condition). Values are expressed as mean ± SD, * *p* < 0.05. (**d**) Survival curves of *DJ-1β* mutant flies cultured in vehicle (0.1% DMSO, Cnt) or treated with 200 µM Pin from larval stages. Survival was analyzed using the Kaplan–Meier method and compared by log-rank. (**e**) Relative ATP levels in 5-day-old *DJ-1β* mutant flies treated with 100 or 200 μM Pin compared to controls of the same age. (**f**) Glycogen levels in 5-day-old *DJ-1β* mutant flies treated with 100 or 200 μM Pin or cultured in vehicle (0.1% DMSO, Cnt) from larval stages. Results are normalized to data obtained in control flies, * *p* < 0.05.

To explore the therapeutic potential of Pin, *DJ-1β* mutants were raised on Pin-supplemented food throughout development, from larval stage to five days after eclosion. Two concentrations of Pin (100 and 200 µM) were tested, and locomotor activity was assessed in 5-day-old adults using climbing assays. Pin treatment significantly improved motor performance at the higher concentration tested (Figure 8a).

*DJ-1β* mutant flies also display increased ROS production, leading to oxidative damage of cellular macromolecules [46] and increased protein carbonylation, a common marker of oxidative protein damage. To determine whether Pin could reduce oxidative protein modifications, carbonyl content was measured in extracts from 5-day-old flies treated with vehicle or Pin. In line with the locomotor data, flies treated at 100 and 200 µM exhibited a significant decrease in protein carbonyl levels (Figure 8b), in agreement with the reduction of ROS levels observed in the yeast PD model (Figure 7b), suggesting that Pin mitigates oxidative protein damage, contributing to improved motor function. However, although oxidative protein damage was reduced at both concentrations, the decrease achieved at 100 µM was likely insufficient to reach the threshold required for functional recovery at the organismal level. Thus, a more pronounced attenuation of oxidative stress, as achieved at 200 µM, is necessary to translate molecular protection into measurable improvements in motor performance. Collectively, the reduction of ROS in yeast and oxidative protein damage *in Drosophila* provides converging evidence that attenuation of oxidative stress may contribute to the protective effects of Pin against PD-related phenotypes.

To further assess the efficacy of Pin during adulthood, a stage more relevant to the clinical onset of PD, flies were treated with 200 µM Pin in the larval stage and climbing assays were conducted both at 5 (as in Figure 8a) and 15 days post-eclosion. Pin significantly enhanced locomotor performance at both time points (Figure 8c), indicating sustained benefits both at early and late stages of the disease, consistent with the progressive nature of PD pathology. Despite this effect, 200 µM Pin did not significantly increase fly lifespan (Figure 8d), indicating a potential enhancement of health span rather than longevity. In conclusion, the functional improvements observed in *DJ-1β* mutant flies indicate that Pin can effectively mitigate deficits resulting from *DJ-1β* dysfunction, ameliorate PD-related phenotypes, and highlight its potential as a therapeutic intervention.

Alterations in cellular energy metabolism are central features of neurodegenerative diseases, including PD. Mitochondrial dysfunction, oxidative stress, and impaired glucose utilization contribute to reducing ATP production, compromising neuronal function, and survival. In this context, astrocytic glycogen has emerged as an important regulator of neuronal energy homeostasis: its mobilization during metabolic, hypoxic, or oxidative stress helps sustain ATP levels and supports synaptic function, plasticity, and overall neuronal resilience [49]. Evidence from *Drosophila* and in vitro PD models suggests that defects in glycolysis and mitochondrial function can activate compensatory mechanisms, including increased glycolytic flux and glycogen mobilization, to sustain energy demands. Accordingly, interventions that enhance ATP availability and glycogen storage could provide neuroprotective benefits by restoring energy balance, reducing oxidative damage, and supporting neuronal survival. These observations underscore the potential of targeting cellular energy metabolism, particularly ATP generation and glycogen dynamics, as a strategy to counteract neurodegenerative processes [27,49].

To investigate the metabolic effects of Pin, the ATP and glycogen levels were measured in 5-day-old female *DJ-1β* mutant flies treated with 100 and 200 µM of Pin. Interestingly, the treatment at both concentrations resulted in a dose-dependent increase in ATP levels, highlighting that Pin improves energy metabolism in the mutant flies, significantly enhancing cellular energy content (Figure 8e). Moreover, a significant increase in glycogen content, a marker of energy reserves [50], was observed at higher concentration (200 µM) (Figure 8f), indicating that Pin also promotes the accumulation of energy reserves. These results indicate that Pin exerts a beneficial influence on energy homeostasis by acting as a modulator of cellular metabolism and intracellular energy reserves.

## 4. Conclusions

This study demonstrates that SFE-CO_2_ represents an effective and sustainable strategy for the valorization of underutilized *G. glabra*, consistent with sustainable resource management and circular bioeconomy. The optimized SFE-CO_2_ enabled the selective recovery and enrichment of leaf-surface flavanones Pin, Lic, and Gla, yielding high-purity, solvent-free extracts with lower environmental impact than conventional solvent-based methods, as confirmed by the Path2Green assessment. This approach supports industrial scalability and may enhance the value chain of PDO Calabrian licorice, although dedicated techno-economic studies will be required to define its industrial feasibility.

Importantly, the SFE-CO_2_ extract displayed enhanced antioxidant capacity in complementary ABTS and ORAC assays and preserved the antioxidant activity previously associated with GGL, reducing oxidative stress and α-syn-induced toxicity in the yeast *S. cerevisiae* PD model. Among the major constituents, pinocembrin emerged as a key bioactive compound, displaying antioxidant and anti-aggregant properties. Moreover, in a *Drosophila melanogaster* PD model, pinocembrin improved locomotor performance, reduced oxidative protein damage, and enhanced ATP production and glycogen storage, supporting a role in maintaining cellular energy homeostasis.

Beyond their biological relevance, these findings provide a useful reference for the development of selective flavanone extraction processes, particularly considering the increasing interest in natural flavonoids and flavanone-based compounds as bioactive templates for pharmaceutical and nutraceutical applications [51].

Overall, these findings highlight SFE-CO_2_ as a green technology with potential for the scalable production of bioactive-enriched extracts with applications in functional foods and neuroprotective nutraceuticals, while promoting the sustainable valorization of agricultural by-products.

## Figures and Tables

**Table 1:** Extraction performance of optimized SFE-CO_2_ compared with conventional solvent-based extraction techniques ^1^.

	SFE-CO_2_	Chloroform-SLE	Hexane-SLE
Extract yield (g 100 g^−1^ leaf)	3.9 ± 0.9 ^c^	34.9 ± 1.9 ^a^	7.8 ± 0.1 ^b^
Fs recovery (% *w*/*w*) ^2^	26.4 ± 4.5 ^b^	79.7 ± 4.7 ^a^	4.1 ± 0.9 ^c^
Pin extract content (% *w*/*w*)	21.8 ± 1.9 ^a^	4.9 ± 0.1 ^b^	0.8 ± 0.1 ^c^
Lic extract content (% *w*/*w*)	4.5 ± 1.1 ^a^	4.4 ± 0.1 ^a^	0.6 ± 0.0 ^b^
Gla extract content (% *w*/*w*)	3.4 ± 0.8 ^a^	0.8 ± 0.0 ^b^	0.4 ± 0.0 ^b^
Fs extract content (% *w*/*w*)	30.5 ± 2.3 ^a^	10.1 ± 0.1 ^b^	1.9 ± 0.1 ^c^
ABTS (mmol TE g^−1^ extract)	4.4 ± 0.1 ^a^	3.1 ± 0.3 ^b^	nd
ORAC (mmol TE g^−1^ extract)	6.7 ± 0.4 ^a^	5.3 ± 1.2 ^b^	nd

^1^ Different superscript letters within the same row indicate significantly different values (*p* ≤ 0.05 by a Tukey’s HSD test); ^2^ determined relative to exhaustive extraction; nd, not determined.

## Data Availability

The original contributions presented in this study are included in the Article/Supplementary Material. Further inquiries can be directed to the corresponding author.

## Supplementary Materials

The following supporting information can be downloaded at: https://www.mdpi.com/article/10.3390/antiox15070874/s1, Table S1: BBD matrix, target response values and predicted and experimental results obtained under optimal SFE-CO_2_ conditions.; Table S2: Analysis of Variance (ANOVA) for response variables of SFE-CO_2_ extraction; Table S3: Regression equations of the fitted models and corresponding optimal levels of SFE-CO_2_ factors; Table S4: Detailed scores of the 12 green extraction principles obtained using the Path2Green metric; Figure S1: Response surfaces of (a) extraction efficiency of Lic and Gla, (b) extract content of Lic, and (c) extraction yield as a function of pressure and dynamic time (on the left, temperature fixed at 60 °C) and of temperature and pressure (on the right, dynamic time fixed at 60 min; Figure S2: UHPLC–UV profiles at 290 nm of SFE-CO_2_ extract obtained at 40 °C, 364 bar, 30 min (dark green line) and at 40 °C, 370 bar, 90 min (light green line); Figure S3: Desirability plot of SFE-CO_2_ optimization.
