# Supplementary material for: Selective Recovery of Flavanone-Enriched Fractions from Glycyrrhiza Glabra Leaves by Supercritical CO2 Extraction with Neuroprotective Potential
Source: Antioxidants (Basel). 2026 Jul 14;15(7):874. doi: 10.3390/antiox15070874 (PMC13404160; doi:10.3390/antiox15070874)

Supplementary material

Selective Recovery of Flavanone-Enriched Fractions from *Glycyrrhiza glabra* Leaves by Supercritical CO_2 Extraction with Neuroprotective Potential

Simona Serio[a,b], Alessia Lambiase[c,d], Valentina Santoro[a,c], Anna Lisa Piccinelli[a,c,*], Rita Celano[a,c], Giorgia Spandri[d], Farida Tripodi[c,d], Luca Campone[c,d], Stefania Pagliari[c,d], Paola Coccetti[c,d], Cristina Solana-Manrique[e,f], Nuria Paricio[e,f], Mariateresa Russo[g], Massimo Labra[c,d], Luca Rastrelli[a,c]

[a]Department of Pharmacy, University of Salerno, Fisciano, Salerno, Italy.

[b]PhD Program in Drug Discovery and Development, University of Salerno, Fisciano, Salerno, Italy.

[c]National Biodiversity Future Center (NBFC), Palermo, Italy.

[d]Department of Biotechnology and Biosciences, University of Milano-Bicocca, Milano, Italy.

[e]Department of Genetics and University Institute of Biotechnology and Biomedicine, University of Valencia, Spain.

[f]Department of Physiotherapy, European University of Valencia, Spain.

[g]Department of Agriculture Science, Food Chemistry, Safety and Sensoromic Laboratory (FoCuSS Lab), University of Reggio Calabria, Via Salita Melissari, 89124 Reggio Calabria, Italy.

Table S1. BBD matrix, target response values and predicted and experimental results obtained under optimal SFE-CO_2 conditions.

**Independent factors**	**Units**	**Level -1**	**Level 0**	**Level 1**
A: Temperature	°C	40	60	80
B: Pressure	bar	150	275	400
C: Dynamic time	min	30	60	90

**Run**	**Block**	**Temperature**	**Pressure**	**Dynamic time**	**EE Pin**	**EE Lic**	**EE Gla**	**EE Tot**	**P Pin**	**P Lic**	**P Gla**	**P Tot**	**Yield**	**CO_2**
		**°C**	**bar**	**min**	**g 100 g-1 leaf**				**g 100g^{-1} extract**				**g 100 g^{-1}**	**L min^{-1}**
1	1	40	150	60	0.37	0.06	0.08	0.5	14.9	2.3	3.4	21.1	2.1	120
2	1	60	275	60	0.82	0.20	0.13	1.2	18.7	4.7	3.0	27.2	4.4	120
3	1	60	275	60	0.78	0.19	0.13	1.3	19.1	4.8	3.3	28.1	4.1	120
4	1	40	275	30	0.63	0.13	0.09	0.9	18.9	3.8	2.7	26.2	3.3	60
5	1	40	275	90	0.96	0.28	0.12	1.4	19.7	5.9	2.4	28.8	4.8	180
6	1	80	275	30	0.64	0.13	0.13	0.9	17.1	3.4	3.5	25	3.8	60
7	1	80	150	60	0.19	0.02	0.06	0.3	8.9	1.0	2.6	13.4	2.2	120
8	1	60	150	90	0.33	0.05	0.08	0.5	12.5	1.9	3.0	18.2	2.7	180
9	1	60	150	30	0.15	0.02	0.05	0.2	12.5	1.3	3.7	18.5	1.2	60
10	1	60	400	90	0.93	0.33	0.12	1.4	18.9	6.7	2.5	28.9	4.9	180
11	1	40	400	60	0.90	0.30	0.12	1.4	18.7	6.3	2.5	28.3	4.8	120
12	1	60	275	60	0.85	0.23	0.13	1.3	17.7	4.7	2.6	25.8	4.8	120
13	1	80	275	90	0.83	0.23	0.15	1.3	14.5	4.1	2.7	22.1	5.7	180
14	1	60	400	30	0.72	0.15	0.13	1.0	20.8	4.4	3.7	29.9	3.5	60
15	1	80	400	60	0.84	0.28	0.15	1.3	15.5	5.2	2.7	24.1	5.4	120
16	1	60	275	60	0.74	0.20	0.12	1.1	16.1	4.3	2.6	23.8	4.6	120
**Optimum**		(EE Tot, P Tot, CO_2 consumption)			**EE Pin**	**EE Lic**	**EE Gla**	**EE Tot**	**P Pin**	**P Lic**	**P Gla**	**P Tot**	**Yield**	**CO_2**
Predicted values		**40**	**364**	**30**	0.8	0.2	0.1	1.1	20.7	5.0	2.9	30.3	4.0	60.0
95% confidence interval					0.7 - 0.8	0.1 - 0.2	0.1 - 0.1	1.0 - 1.2	19.3 - 22.2	4.6 - 5.5	2.0 - 3.8	28.4 - 32.2	3.6 - 4.5	60.0 - 60.0
Desirability			**0.92**		0.8	0.5	0.6	0.7	1.0	0.7	0.5	1.0	0.6	1.0
Experimental value (mean ± SD. *n* = 4)					0.8 ± 0.1	0.2 ± 0.1	0.1 ± 0.0	1.2 ± 0.2	21.8 ± 1.9	4.5 ± 1.1	3.4 ± 0.8	30.5 ± 2.3	3.9 ± 0.9	60.0 ± 0.0
95% confidence interval					0.7 - 0.9	0.1 - 0.3	0.1 - 0.1	0.9 - 1.3	20.6 - 24.0	3.7 - 5.7	2.8 - 4.3	28.3 - 33.0	2.8 - 4.4	60.0 - 60.0

Table S2. Analysis of Variance (ANOVA) for response variables of SFE-CO_2 extraction.

	**Pin (% leaf)**		**Lic (% leaf)**		**Gla (% leaf)**		**Fs (% leaf)**		**Pin (% extract)**		**Lic (% extract)**		**Gla (% extract)**		**Fs (% extract)**		**Extraction yield**	
	F-value	*p*-value	*F*-value	*p*-value	*F*-value	*p*-value	*F*-value	*p*-value	*F*-value	*p*-value	*F*-value	*p*-value	*F*-value	*p*-value	*F*-value	*p*-value	*F*-value	*p*-value
**A: Temperature**	7.1	0.0764	5.04	0.1104	32.00	0.0109	3.45	0.1601	18.34	0.0234	54.03	0.0052	0.33	0.6078	13.94	0.0335	6.18	0.0888
**B: Pressure**	301.2	0.0004	345.04	0.0003	312.50	0.0004	341.47	0.0003	44.11	0.0070	658.19	0.0001	1.69	0.2849	57.05	0.0048	151.63	0.0012
**C: Dyn. time**	45.2	0.0067	88.17	0.0026	24.50	0.0158	59.95	0.0045	0.96	0.3999	82.60	0.0028	9.71	0.0526	0.09	0.7824	55.64	0.0050
**AA**	0.1	0.8479	0.75	0.4502	0.25	0.6514	0.13	0.7393	2.30	0.2266	0.79	0.4387	2.05	0.2475	2.32	0.2249	2.53	0.2099
**AB**	1.6	0.2989	0.33	0.6042	25.00	0.0154	1.92	0.2595	1.09	0.3733	0.22	0.6682	1.95	0.2572	0.86	0.4215	0.70	0.4639
**AC**	2.1	0.2398	2.08	0.2446	1.00	0.3910	2.35	0.2228	1.62	0.2931	9.97	0.0510	0.54	0.5158	2.16	0.2384	0.45	0.5509
**BB**	90.3	0.0025	30.08	0.0119	110.25	0.0018	83.26	0.0028	12.77	0.0374	55.21	0.0050	0.84	0.4262	10.80	0.0464	53.05	0.0053
**BC**	0.1	0.7745	18.75	0.0227	16.00	0.0280	0.90	0.4126	0.51	0.5285	14.69	0.0313	0.54	0.5158	0.03	0.8637	0.03	0.8777
**CC**	2.5	0.2152	5.33	0.1041	6.25	0.0877	3.88	0.1433	0.99	0.3938	4.16	0.1340	1.30	0.3376	0.60	0.4939	4.38	0.1274
**Lack of fit**	0.57	0.6717	1.36	0.4030	7.67	0.0642	0.91	0.5289	0.54	0.6866	0.12	0.9442	1.23	0.4339	0.41	0.7599	1.26	0.43
**Lack of fit [a]**	0.34	0.8743	1.69	0.2559	5.08	0.1050	0.74	0.6162	1.38	0.3360	2.21	0.2758	-	-	1.31	0.3588	1.28	0.3707
**Model**		0.0000		0.0000		0.0001		0.0000		0.0000		0.0000		0.2267		0.0000		0.0000
**R^2**		98.96		98.59		95.30		98.86		94.70		99.62		73.88		95.41		97.59
**adjusted R^2**		97.41		96.48		88.26		97.15		86.76		99.05		34.71		88.54		93.97
**R^2 [a]**		96.02		95.90		93.95		96.35		86.16		97.91		-		88.82		92.52
**adjusted R^2 [a]**		95.03		94.41		89.92		95.43		82.70		96.86		-		86.03		90.65

[a] not significant terms excluded ($p > 0.05$).

Table S3. Regression equations of the fitted models and corresponding optimal levels of SFE-CO_2 factors.

**Response variables (Y)**	**Regression equation**	**Optimal factors**		
		**A**	**B**	**C**
**Pin (g/100 g leaf)**	$-1.1936 + 0.010358\times B + 0.00379167\times C - 0.00001456\times B^2$	58	356	90
**Lic (g/100 g leaf)**	$-0.2314 + 0.001982\times B - 0.000833333\times C - 0.00000304\times B^2 + 0.00001\times B\times C$	60	400	90
**Gla (g/100 g leaf)**	$-0.0798 - 0.000875\times A + 0.001034\times B + 0.001025\times C + 0.000005\times A\times B - 0.00000168\times B^2 - 0.00000266667\times B\times C$	80	355	90
**Tot (g/100 g leaf)**	$-1.727 + 0.01458\times B + 0.00625\times C - 0.00002\times B^2$	44	364	90
**Pin (g/100 g extract)**	$5.32945 - 0.101188\times A + 0.109194\times B - 0.00015288\times B^2$	40	357	65
**Lic (g/100 g extract)**	$-1.77545 - 0.0288125\times A + 0.038286\times B - 0.00741667\times C - 0.00005272\times B^2 + 0.000113333\times B\times C$	40	400	90
**Tot (g/100 g extract)**	$7.3919 - 0.123625\times A + 0.148348\times B - 0.00019696\times B^2$	40	377	58
**Yield (g/100 g)**	$-5.261 + 0.04868\times B + 0.02625\times C - 0.0000696\times B^2$	46	350	90

Table S4. Detailed scores of the 12 green extraction principles obtained using the Path2Green metric.

Green Extraction Principles	Weight	Score (lab scale)	Score (large scale)
1. Biomass: Select biomass that is naturally sourced or requires minimal resource usage for production	6.0	+ 1 (waste)	+ 1 (waste)
2. Transport: Preserving biomass integrity while minimizing transport's environmental impact	5.0	0.05 (265 km)	+1 (0 km)
3. Pre-treatment: Pre-treatment: Optimization for pre-treatment avoidance and cost-effective techniques	2.5	- 0.2 (physical pretreatment)	- 0.2 (physical pretreatment)
4. Solvent: Minimize solvent usage, prioritizing those of biological origin, biodegradable and non-toxic	6.0	+ 1 (CHEM21)	+ 1 (CHEM21)
5. Scaling: Ensure reproducibility and a continuous extraction flow	5.0	+ 0.5 (Semi-continuous)	- 1 (In batches)
6. Purification: Final application dictates the extent of purification	2.5	+ 1 (ready to use)	+ 1 (ready to use)
7. Yield: Maximize the utilization and valorization of the biomass	4.0	- 0.50 (semi-exhaustive)	+ 1 (full valorization)
8. Post-treatment: Functionalization of natural products post-extraction to maximize their benefits	2.5	+ 1 (ready to use)	+ 1 (ready to use)
9. Energy: Prioritize using clean energy sources and high-efficiency extraction techniques	5.0	- 0.50 (high dependent/renewable)	- 0.50 (high dependent/renewable)
10. Application: Ensure safety for applications in several domains	4.5	+ 0.83 (at last 5 domains)	+ 0.83 (at last 5 domains)
11. Repurposing: Trace strategies to perform closed-loop extraction systems, preferably using non-virgin materials	6.0	0 (raw material)	0 (raw material)
12. Waste management: Refine waste reduction and ensure effective waste management	6.0	- 0.9 (95%)	+ 1 (full reuse)

Figure S1. Response surfaces of (a) extraction efficiency of Lic and Gla, (b) extract content of Lic and (c) extraction yield as a function of pressure and dynamic time (on the left, temperature fixed at 60 °C) and of temperature and pressure (on the right, dynamic time fixed at 60 min). Only statistically significant effects are shown.

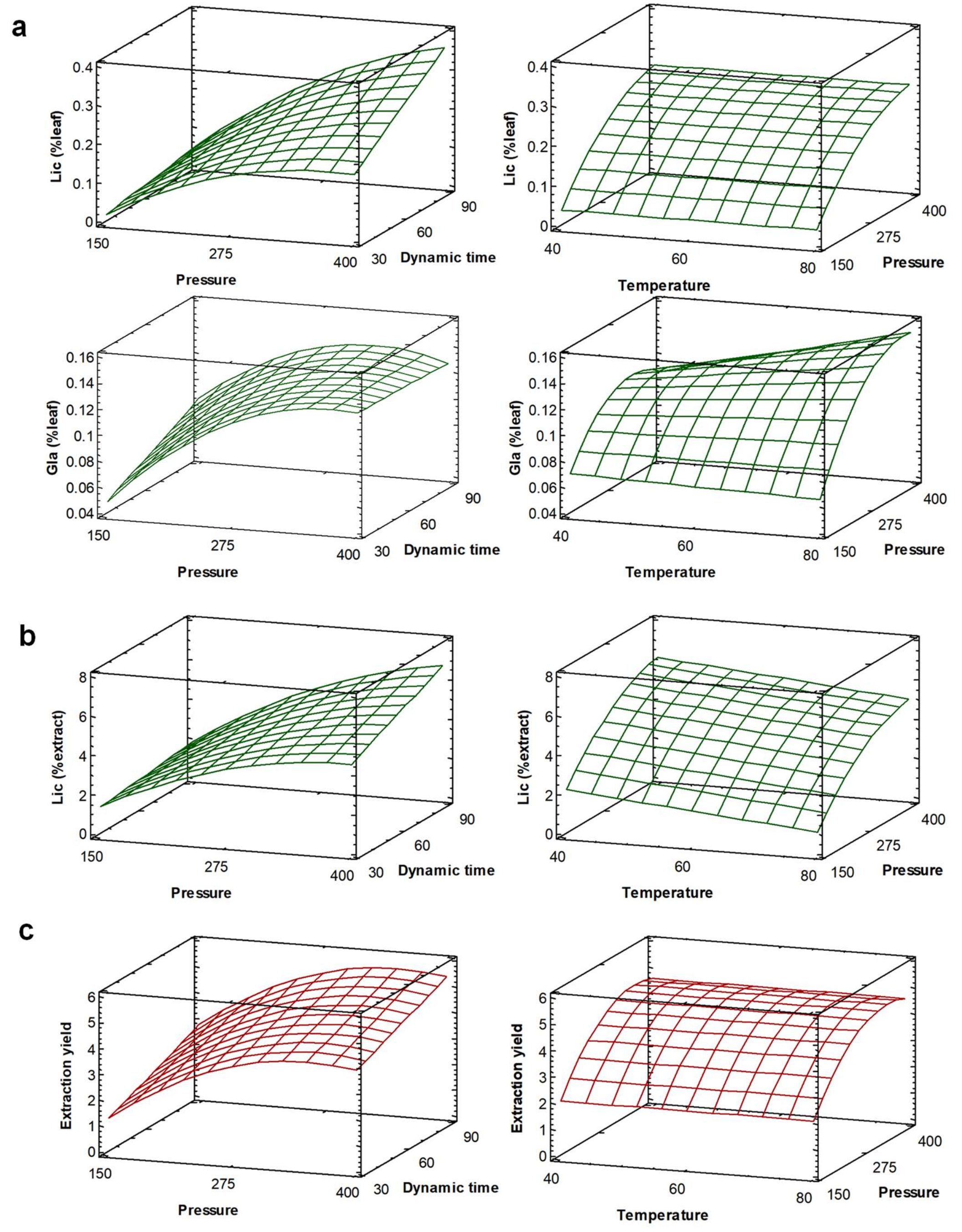

Figure S2. UHPLC-UV profiles at 290 nm of SFE-CO_2 extract obtained at 40°C, 364 bar, 30 min (dark green line) and at 40°C, 370 bar, 90 min (light green line).

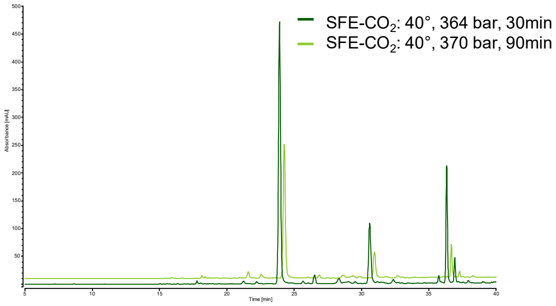

Figure S3. Desirability plot of SFE-CO_2 optimization.

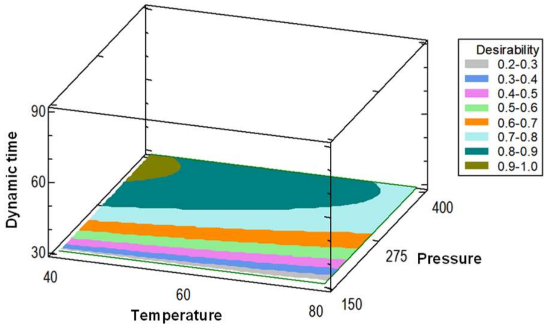

Supplement: Supplementary file 1 [file antioxidants-15-00874-s001.zip › antioxidants-4415727-supplementary.pdf]
